# Unveiling the Emerging Role of Extracellular Vesicle–Inflammasomes in Hyperoxia-Induced Neonatal Lung and Brain Injury

**DOI:** 10.3390/cells13242094

**Published:** 2024-12-18

**Authors:** Karen Young, Merline Benny, Augusto Schmidt, Shu Wu

**Affiliations:** Division of Neonatology, Department of Pediatrics, Batchelor Children Research Institute, University of Miami School of Medicine, Miami, FL 33136, USA; kyoung3@med.miami.edu (K.Y.); mxk968@med.miami.edu (M.B.); aschmidt@med.miami.edu (A.S.)

**Keywords:** pre-term infant, BPD, brain injury, extracellular vesicles, inflammasomes

## Abstract

Extremely premature infants are at significant risk for developing bronchopulmonary dysplasia (BPD) and neurodevelopmental impairment (NDI). Although BPD is a predictor of poor neurodevelopmental outcomes, it is currently unknown how BPD contributes to brain injury and long-term NDI in pre-term infants. Extracellular vesicles (EVs) are small, membrane-bound structures released from cells into the surrounding environment. EVs are involved in inter-organ communication in diverse pathological processes. Inflammasomes are large, multiprotein complexes that are part of the innate immune system and are responsible for triggering inflammatory responses and cell death. Apoptosis-associated speck-like protein containing a caspase recruitment domain (ASC) is pivotal in inflammasome assembly and activating inflammatory caspase-1. Activated caspase-1 cleaves gasdermin D (GSDMD) to release a 30 kD N-terminal domain that can form membrane pores, leading to lytic cell death, also known as pyroptosis. Activated caspase-1 can also cleave pro-IL-1β and pro-IL-18 to their active forms, which can be rapidly released through the GSDMD pores to induce inflammation. Recent evidence has emerged that activation of inflammasomes is associated with neonatal lung and brain injury, and inhibition of inflammasomes reduces hyperoxia-induced neonatal lung and brain injury. Additionally, multiple studies have demonstrated that hyperoxia stimulates the release of lung-derived EVs that contain inflammasome cargos. Adoptive transfer of these EVs into the circulation of normal neonatal mice and rats induces brain inflammatory injury. This review focuses on EV–inflammasomes’ roles in mediating lung-to-brain crosstalk via EV-dependent and EV-independent mechanisms critical in BPD, brain injury, and NDI pathogenesis. EV–inflammasomes will be discussed as potential therapeutic targets for neonatal lung and brain injury.

## 1. Introduction

More than 15 million infants are born pre-term each year worldwide [1,2,3]. Extremely premature infants born at less than 28 weeks of gestational age (GA) are at significant risk of having multi-organ injury and developmental abnormalities that predominately involve the lungs and brain. The lungs of these infants are immature, and they suffer respiratory failure soon after birth and often require supplemental oxygen (O_2_) [2,3]. However, life-sustaining high fraction inspired O_2_ (FiO_2_) can cause lung inflammation, leading to damage of lung structures and, ultimately, bronchopulmonary dysplasia (BPD) [2,3]. These infants’ brains are also immature and predisposed to injurious stimuli such as oxygen toxicity and inflammation. Therefore, they are at increased risk of developing short-term and long-term neurological complications [4,5,6]. Thus, BPD survivors not only have pulmonary dysfunction but often also suffer from long-term neurodevelopmental impairment (NDI) [6,7]. As such, the cost of treating BPD is USD 2.4 billion/year [8], and for early intervention is >USD 1.3 billion/year in the US [6,7,9,10]. There is mounting clinical evidence that severe BPD is an independent risk factor for brain injury and NDI [6,7,11]. Evidence from preclinical studies demonstrates that hyperoxia exposure induces BPD-like pathology and results in brain injury and NDI in newborn rodents [12,13,14,15]. Despite recent advances in neonatal intensive care and extensive research, it is unknown to what extent BPD contributes to NDI, and there is no effective therapy for either of these conditions. Thus, understanding the mechanisms by which BPD contributes to NDI and identifying novel therapeutic targets for these two severe complications of prematurity are paramount. This review focuses on circulating extracellular vesicle (EV)–inflammasomes’ roles in mediating lung-to-brain crosstalk via EV-dependent and EV-independent mechanisms critical in BPD and brain injury pathogenesis. EV–inflammasomes will be discussed as potential therapeutic targets for neonatal lung and brain injury. 

## 2. BPD Is Associated with NDI: Clinical Evidence

BPD was first described by Northway, et al. in 1967 as a chronic lung disease of pre-term infants who had hyaline membrane disease and required high supplemental oxygen therapy and mechanical ventilation [16]. The infants who developed BPD were moderate to late pre-term, and their lungs had significant structural damage, the so-called “old” or “classic” BPD [16]. Over the last 50 years, the incidence of BPD has not decreased because the survival rate of extremely low gestational age newborns (ELGANs) born at <28 weeks GA has increased [17]. The incidence of BPD is inversely related to the GA, and the so-called “new” BPD presents with distinct clinical and pathological characteristics due to the advanced neonatal intensive care. The hallmarks of the current BPD pathology are alveolar simplification, poor vascular growth, and chronic inflammation [2]. In 2015, it was reported that BPD occurs in ~35% of ELGANs in the USA, as almost 18,000 out of 50,000 such infants developed BPD [18]. However, a wide range of BPD prevalences, from 11 to 50%, have been demonstrated by major neonatal research networks worldwide [19,20,21,22,23,24,25], which may be secondary to the non-uniform use of the BPD definitions. The most widely used BPD definition was proposed by the National Institute of Child Health and Human Development (NICHD)/National Heart Lung and Blood Institute (NHLBI) workshop in 2001 (Table 1). This definition was based on the levels of supplemental oxygen and the mode of respiratory support at 36 weeks postmenstrual age (PMA) in pre-term infants born at <32 weeks GA who required oxygen therapy at 28 days of life [2]. The Canadian Neonatal Research Network suggested that evaluating infants at 40 weeks’ PMA may be more predictive of respiratory outcomes in 2017 (Table 1) [22]. In 2018, the NICHD workshop proposed a more complex definition that combines oxygen therapy with the mode of respiratory support at 36 weeks PMA. The workshop also proposed to use terms of grades I, II, and III rather than mild, moderate, and severe, as proposed in the 2001 workshop (Table 1) [26]. In 2019, NICHD proposed an updated definition of BPD with severity stratification solely based on the mode of respiratory support at 36 weeks PMA, regardless of supplemental oxygen use (Table 1) [27]. Regardless of what definition is used, the pathological hallmarks of BPD are well known, including alveolar simplification, poor vascularization, variable fibrosis, and chronic inflammation [2,28]. However, the pathogenesis of BPD is poorly understood, and there is no effective therapy for this common complication of prematurity. 

There is mounting evidence that pre-term infants who develop BPD are at increased risk for brain injury and NDI [29,30,31]. BPD survivors exhibit various degrees of impairment in motor, neurosensory, general cognitive functioning, attention, language, memory and learning, visual-spatial perception, executive skills, academic performance, and psychological problems. Cerebral palsy (CP) occurs in approximately 10% of pre-term infants born at <28 weeks GA [32,33]. However, the incidence of CP is even higher at 15% in the BPD survivors [33]. One study showed that BPD was a significant risk factor for CP at 18–22 months corrected age (CA) (odds ratio 1.66, 95% confidence interval 1.01 to 2.74) after adjusting for other confounding factors such as cystic periventricular leukomalacia (PVL) and severe intraventricular hemorrhage (IVH) [34]. At 24 months of age, BPD requiring mechanical ventilation (BPD/MV) at 36 weeks PMA was associated with a nearly sixfold increased risk of quadriparesis and a fourfold increased risk of diparesis [31]. Other non-CP motor impairments also occur more frequently in children who had BPD at 8 years old [35,36,37].

Gallini et al. demonstrated that not only infants with severe BPD, but also those with moderate BPD, had an increased risk of overall cognitive impairment at 20 months CA [38]. In a study of pre-term infants at 18 months CA, BPD was an independent risk factor for major neurosensory problems with an OR (95% CI) of 2.4 for any of CP, blindness, deafness, or cognitive delay [39]. A similar study showed that at 5 years of age, children with a history of BPD had a higher chance of disability in one or more areas of motor impairment, cognitive impairment, behavior problems, poor general health, deafness, or blindness [40]. A recent study found that increased BPD severity is associated with increased risk of NDI at both 2 years and 5 years CA [41] assessed by Bayley Scales of Infant and Toddler Development (BSID)-II or BSID-III at 2 years of age [41,42,43] and Wechsler Preschool and Primary Scale of Intelligence at 5 years CA [44]. In a study with children at 8 years old, BPD survivors had the worst performance in general intelligence, reading, mathematics, motor performance, memorization, and attention [36]. Children who had more severe BPD compared with children who had mild/moderate BPD performed worse in performance IQ and perceptual organization at 3 and 8 years of age [45]. Some studies have reported language delay in children with BPD. Extremely low birth weight children with a history of BPD had a significant language delay at 18–22 months compared to children without BPD [46]. In a study of 3-year-old very low birth weight preschool children, the group with BPD had lower scores in receptive, expressive, and total communicative competence domains [34]. When followed up at 8 years old, children with BPD had stepwise decreases in scores for both expressive and receptive language compared to children without BPD [47].

Children with BPD also have impaired visual-spatial perception compared to children without BPD at 8–10 years old [48]. Taylor et al. demonstrated that a longer duration of oxygen therapy to treat BPD is associated with difficulties in perceptual-motor tasks in very low birth weight children aged 7 years [49] and also at 16 years of age [49,50]. A recent study in children 5–6 years of age found that BPD was strongly associated with mild, moderate, and severe overall neurodevelopmental disabilities (OR 1.49, 95% CI 1.05 to 2.20; 2.20, 1.41 to 3.42 and 2.71, 1.67 to 4.40) [7]. BPD was also associated with developmental coordination disorders, behavioral difficulties, lower IQ scores, rehospitalization in the last 12 months, and developmental support [7]. A recent study comparing BPD with and without tracheostomy found that BPD with tracheostomy had greater cognitive and motor delays <24 months and more significant cognitive delays >24 months of age [51]. This study was conducted at 2 years CA in pre-term infants with various degrees of BPD, as defined by Jensen et al. [27]. Grade 1 BPD is defined as receiving nasal cannula with a flow rate of 2 L per minute (LPM) or less, grade 2 BPD is defined as receiving nasal cannula of more than 2 LPM flow rate or noninvasive positive pressure support, and grade 3 is defined as receiving invasive respiratory support. Grades 2 and 3 BPD, but not grade 1, were found to be associated with increased odds of a composite adverse neurodevelopmental outcome by 2.7- and 7.2-fold, respectively. A BSID domain-specific analysis showed that higher grades of BPD were associated with lower scores in the cognitive, gross motor, and fine motor domains [9]. Infants with grade 3 BPD had increased odds of CP and developmental delay at 2 years of age, poor academic achievement, and low IQ in adolescence [52].

These data demonstrate that survivors of infants with BPD, particularly those with more severe lung disease in the neonatal period, are at increased risk for NDI throughout childhood. The underlying mechanisms for BPD contributing to NDI remain to be explored. 

## 3. Animal Models of Hyperoxia-Induced BPD and Brain Injury

**Figure 1:**
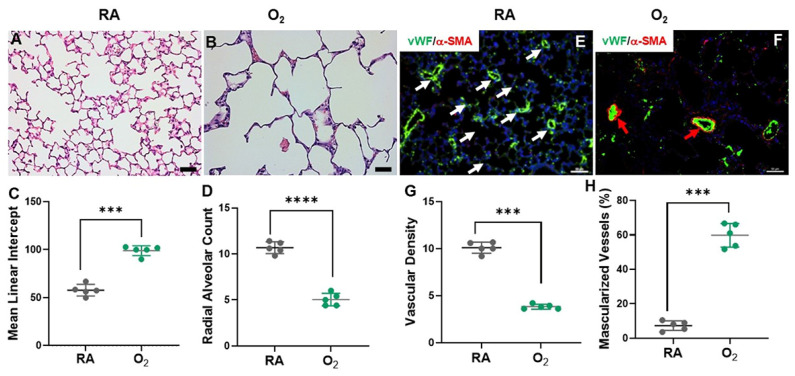
Mouse model of hyperoxia-induced BPD. Newborn mice were exposed to room air (RA) or 85% O_2_ from P1 to P14. (**A**,**B**): Lung morphology was assessed by mean linear intercept (MLI) (**C**) and radial alveolar count (RAC) (**D**). Hyperoxia exposure increased MLI and decreased RAC, suggesting impaired alveolarization. (**E**,**F**): immunostaining for vWF (white arrows) and alpha-smooth muscle actin (α-SMA) (red arrows). Hyperoxia exposure reduced vWF+ vessel counts (**G**) and increased α-SMA positive vessels (**H**). Magnification: 20×. Scale bar: 50 μm. *** *p* < 0.001. **** *p* < 0.0001. Ref. [55] with permission.

There are many studies using animal models of hyperoxia-induced neonatal lung and brain injury to mimic BPD and brain injury in pre-term infants. It is important to note that while these animal models provide valuable insights, they are not perfect replicas of human conditions and have their limitations. The lung development goes through embryonic [embryonic days (E) 9.5–12.5], pseudoglandular (E12.5–16.5), canalicular (E16.5–17.5), saccular [E17.5-postnatal day (P) 5], and alveolar (P0–28) stages in mice [53]. The stages of rat lung development are similar to mouse lung development. Thus, the newborn rodent’s lungs are structurally comparable to the lungs of pre-term infants born at 23–32 weeks of gestation age who are at risk for developing BPD. Exposure of newborn mice and rats to hyperoxia induces the hallmarks of BPD, as demonstrated by alveolar simplification, dysmorphic angiogenesis, and vascular remodeling (Figure 1) [28,54,55]. These phenotypical changes are associated with increased cell death, decreased cell proliferation, elevated inflammatory responses, reduced production of surfactant-associated proteins, and profibrotic changes in the extracellular matrix [28,56,57]. Hyperoxia also induces transcriptomic changes in the BPD models, which are significant as they provide insights into the molecular mechanisms underlying BPD. Xia et al. conducted single-cell RNA-seq (scRNA-seq) in their hyperoxia-induced mouse BPD model. They reported that neonatal hyperoxia-induced inflammation in alveolar type II epithelial (AT2) cells gave rise to damage-associated transient progenitors [58]. Hyperoxia also induced a new subpopulation of alveolar type I epithelial (AT1) cells that have reduced expression of growth factors normally secreted by these cells. The endothelium of hyperoxia-exposed lungs had an expansion of Car4+ endothelial cells (Cap2) along with an emergent Cap2 subpopulation, which had repressed VEGF signaling. The hyperoxia-exposed mesenchymal cells had inflammatory signatures, represented by a new distal interstitial fibroblast subcluster with the characteristics of decreased lipid biosynthesis and a transcriptomic signature resembling myofibroblasts [58]. In another study by Hurskainen et al., hyperoxia exposure from the day of birth to P14 in mice altered the composition of alveolar epithelial cells (AECs), capillary endothelial cells, stromal fibroblasts, and macrophage populations revealed by scRNA-seq at P3, 7, and 14 [59]. They identified that BPD-associated genes were expressed highest in capillary endothelial cells, inflammatory cells (macrophages, neutrophils, nature killer cells, mast and basophil, and T cells), AT1 and AT2 cells, fibroblasts, and mesothelial cells. They further analyzed the signaling pathway and cell communication and found that hyperoxia induces inflammation by activating particular endothelial cells, epithelial cells, and stromal and resident lung immune cells that promote innate and adaptive immune responses [59]. These are just some of the examples that demonstrate hyperoxia-induced mouse models of BPD are associated with wide ranges of transcriptomic changes. 

**Figure 2:**
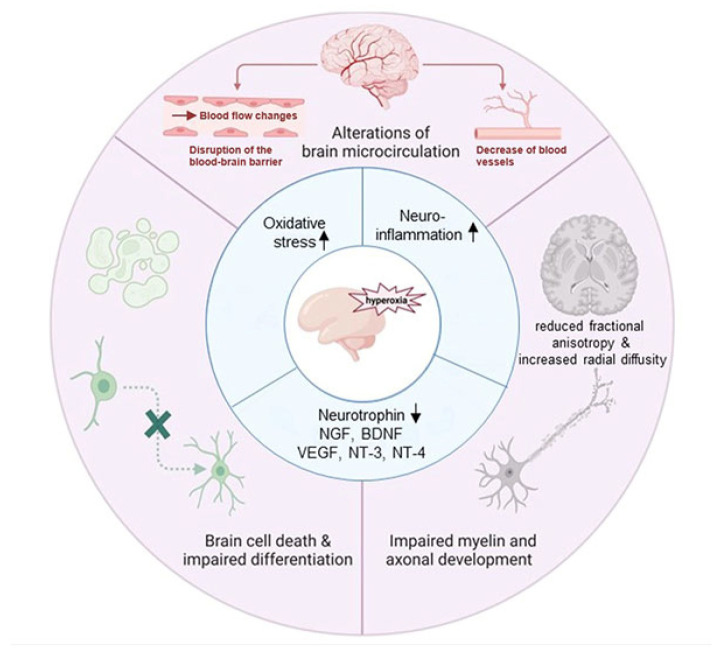
Hyperoxia-induced neonatal brain injury in mice. Hyperoxia increases neuroinflammation and oxidative stress and decreases neurotrophins. These lead to brain cell death and impaired differentiation, altered brain microvascular development, and impaired myelination and axonal development. NGF: nerve growth factor. BDNF: brain-derived neurotrophic factor. VEGF: vascular endothelial growth factor. NT-3: neurotrophin-3. NT-4: neurotrophin-4. Ref. [68] with permission.

Similar to the extensive studies in hyperoxia-induced rodent models of BPD, hyperoxia-induced brain injury is being increasingly investigated. The brain development at P1–3 in rodents is comparable to 23–32 weeks of gestation in humans, and P7–10 in rodents is equivalent to 36–40 weeks of gestation in humans [60,61]. During P1–3, the oligodendrocyte maturation state changes with a predominance of mitotically active pre-oligodendrocytes [61], the immune system develops [62], and the blood–brain barrier (BBB) is established [62]. The neonatal rodent models of hyperoxia-induced brain injury demonstrate increased cell death, oxidative stress, inflammatory responses, reduced neurotrophins, and altered microvascular development in many areas of the developing brains (Figure 2) [13,63,64,65,66,67,68]. Hyperoxia interrupts neuroplasticity, interferes with axonal growth, and causes hypomyelination (Figure 2) [69,70,71]. Felderhoff-Mueser et al. investigated the effects of postnatal hyperoxia on brain injury in rats. They found that hyperoxia exposure (80%) for 24 h in P7 rats increased cell death at the caudate nucleus, nucleus accumbens, layer II and IV of the frontal, parietal, cingulate, retrosplenial cortices, and white matter tracts within the forebrain [67]. They determined that hyperoxia-induced apoptotic cell death is age-dependent by demonstrating that exposure to 80% oxygen for 24 h at P0, 3, 7, 14, and 20, the time window between 0 and 14 days was vulnerable to hyperoxia-induced cell death. On P0, the most affected areas included thalamic nuclei, caudate nucleus, putamen, hypothalamus, and white matter tracks. On P7, the cortical areas were most affected, and the highest overall vulnerability was detected on days 3 and 7. The dentate gyrus was found to have some degenerating cells at P14 [67]. They also found that 40% oxygen did not cause cell death, but 60% oxygen increased apoptotic scores in the rat brain. Hyperoxia also induced inflammatory responses in the developing brain of newborn rats, represented by significantly increased expressions of mRNA and protein levels of caspase-1 and its downstream effectors IL-1β, IL-18, and IL-18 receptor α (IL-18Rα) [72]. White matter injury can be induced by hyperoxia exposure in newborn rodents, demonstrated by increased oligodendrocyte death that resulted in hypomyelination [73], and ultrastructural changes in the white matter with a reduction in myelin thickness, abnormal myeline loops, and decreased axonal caliber [74]. Utilizing genetically modified mouse models, studies have revealed the critical roles of Notch, Ras, and toll-like receptor 4 (TLR4) signaling pathways in hyperoxia-induced neonatal brain injury. One study demonstrated that deficiency of delta-like 4 (DLL4), a Notch signaling ligand, results in persistent microvasculature abnormalities in the developing brain [75]. Interestingly, the inhibition of Notch signaling by a γ-secretase inhibitor significantly attenuated hyperoxia-induced disruption of oligodendrocyte maturation [76]. Serdar et al. showed that neuronal overexpression of the small GTPase Ras reduced apoptosis, protected neuron and oligodendrocyte survival, and improved long-term myelin sheath ultrastructural development [77]. Liu et al. demonstrated that TLR4, a crucial initiator of the inflammatory cascade, was involved in the immune responses in the hyperoxia-exposed neonatal mouse brain; however, TLR4 knockout (KO) reduced neuronal apoptosis and cognitive deficits in hyperoxia-exposed mice [78]. These animal studies highlight the complicities of hyperoxia-induced immature brain injury that has some similarities with brain injury of premature infants who have received high concentrations of oxygen therapy. 

## 4. Extracellular Vesicles in Neonatal Lung and Brain Injury

**Figure 3:**
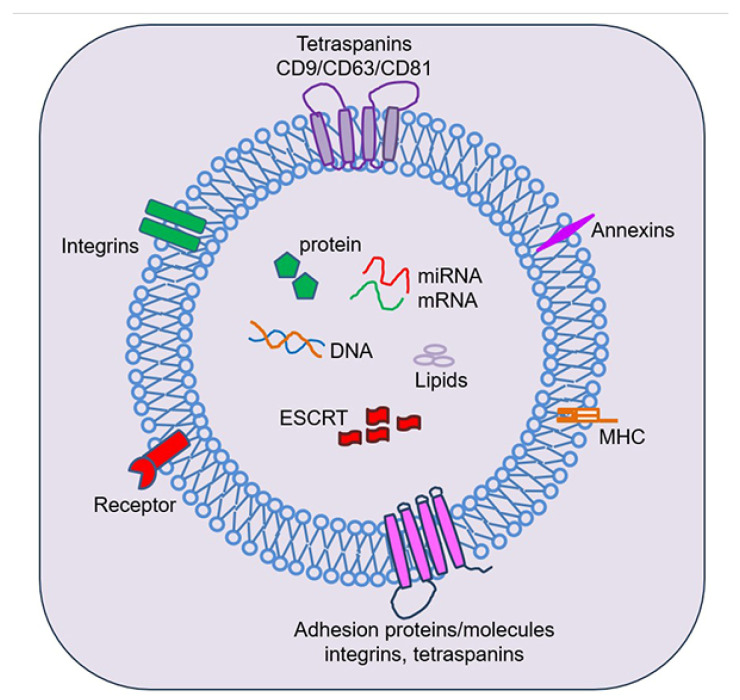
Structure, cargo, and function of extracellular vesicles. Extracellular vesicles (EVs) are composed of a lipid bilayer containing transmembrane proteins with a cargo consisting of proteins, mRNA, miRNA, DNA, and lipids. EVs can be isolated from various body fluids and have a diverse range of sizes ranging from 100 to 1000 nm. EVs isolated from the lung fluids and peripheral blood can be used as biomarkers for neonatal lung diseases. EVs have also been linked to the mediation of neonatal lung disease-associated brain injury.

EVs are lipid-membrane-encircled vesicles secreted by cells into the extracellular environment [79,80,81,82,83,84] (Figure 3). There are three main subtypes of EVs, exosomes, microvesicles, and apoptotic bodies, named based on their biogenesis, release pathways, size, content, and function [79,83,85]. Exosomes are formed by an endosomal route and are sized 30–150 nm in diameter. The direct outward budding of the cell plasma membrane forms microvesicles 100–1000 nm in diameter. Apoptotic bodies are released by dying cells into the extracellular environment, and their size is about 50 nm in diameter. EVs can be detected in many biological fluids, including blood, breast milk, bile, cerebrospinal fluid (CSF), lymph, tracheal aspirate fluid (TAF), urine, and vitreous [86]. With their complex lipids, proteins, and nucleic acid cargoes, EVs are critical players in intercellular and inter-organ communication. Their cargo composition is intricately linked to the biological function of the parental cells. Importantly, being membranous, EVs act as protective carriers, shielding their cargo from the extracellular environment. This ensures the safe transport and delivery of intact cargo to target cells and modifies the target cells’ function. In the lungs, it appears that both AECs and alveolar macrophages (AMs) can release bioactive EVs upon inflammatory injury, as AEC- and AM-derived EVs isolated from TAF have been shown to regulate inflammatory responses in adult lung diseases [87,88,89]. Similarly, EVs have been shown to contribute to several adult central nervous system (CNS) disorders, and it is known that they can bidirectionally cross the BBB [90,91,92]. 

### 4.1. EV Isolation Methods 

There are many methods to isolate EVs from bodily biological fluids, including ultracentrifugation, ultrafiltering, immunoaffinity capture, size-exclusion chromatography, polymer-based precipitation, microfluidics-based techniques, and microchip-based methods. The details about the pros and cons of these methodologies are extensively reviewed [86,93,94,95,96,97] and summarized in Table 2. Ultracentrifugation-based procedures are the gold standard for exosome isolation from cell cultures and distinct body fluids [98]. Isolation of exosomes by ultracentrifugation involves low speed to remove dead cells and cell debris, increasing speed (~10,000) to precipitate larger EVs, such as microvesicles, and followed by high-speed ultracentrifugation (100,100 g) to pellet exosomes [98]. This method is time-consuming and has a high likelihood of contamination by other particles of similar weight and density, and exosome integrity and bioactivity may be damaged due to high shear forces [99]. Ultrafiltration uses varying pore sizes and properties of physical filters to separate exosomes from larger contaminants, including cells, debris, and microparticles [100]. The procedure is faster than ultracentrifugation, and it can also be combined with other techniques, such as ultracentrifugation, size-exclusion chromatography, and low-speed centrifugation [100]. The process is easy to perform, but limitations include low yield, membrane adhesion and blockage, and exosome structural damage. Size-exclusion chromatography is the gentlest procedure frequently used to isolate exosomes based on size. Thus, the isolated exosomes are biologically intact, ideal for subsequent functional research [101]. When combined with ultracentrifugation, the exosome yield and purity are significantly increased [102]. Microfluidic-based techniques are the newest methods for isolating exosomes from small volumes of biological fluids [103]. They offer a relatively simple, low-cost, and continuous particle separation approach and have been well-established for micron-sized particle sorting [103]. Purification methods include membrane-based filtration, acoustic nanofiltration, immunoaffinity, nanowire trapping, deterministic lateral displacement, and viscoelastic flow sorting. There are disadvantages to microfluidic-based techniques, including a lack of standardization of devices, low sampling efficiency, and requirement for high affinity and specificity antibodies [86].

### 4.2. EV Characterization

**Figure 4:**
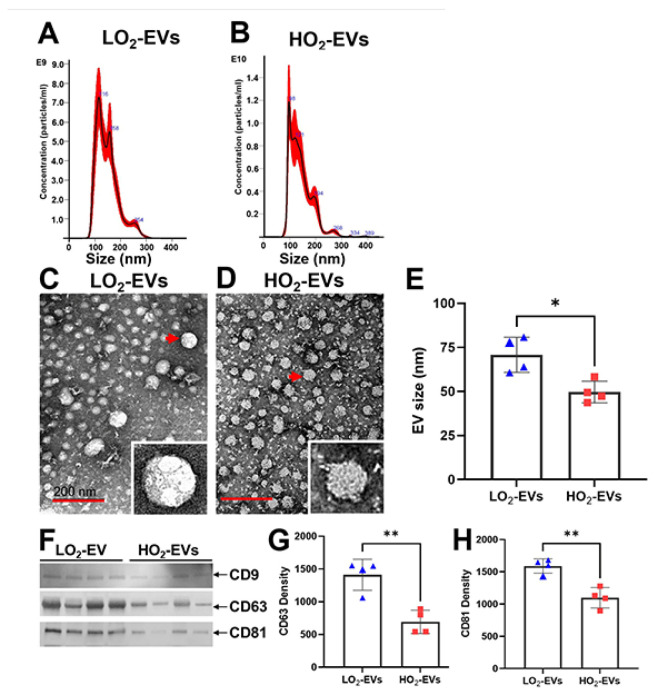
Characterization of EVs. (**A**,**B**): Nanoparticle tracking demonstrates that sizes and concentrations of EVs isolated from the plasma of pre-term infants on lower O_2_ (LO_2_, <30%) and higher O_2_ (HO_2_, >30%) on the seventh day of life. (**C**–**E**): transmission electron microscopy (TEM) shows the EV particles are smaller in size in the HO_2_ group (red arrows). (**F**–**H**): Western blot analysis detects EV surface markers, CD9, CD63, and CD81. The CD63 and CD81 expression levels of the HO_2_ group are lower than the LO_2_ group. * *p* < 0.05, ** *p* < 0.01. Scale bar: 200 nm. Ref. [106] with permission.

The field of EV characterization is significantly important. It focuses on the particle size, shape, density, surface charge, and biocomposition by multiple complementary techniques that are critical for distinguishing EVs from other co-isolated non-EV materials [83]. Based on the 2018 guidelines from The International Society for Extracellular Vesicles Study (MISEV2018), the source of the EV must be described quantitatively, and global quantification of EVs should be provided [83]. MISEV2018 also advocates using single particle analysis techniques that estimate the biophysical features of EVs, demonstrating single EV images at high resolution by electron microscopy, and assessing the topology of EV-associated components [83]. As with EV isolation, EV characterization is also extensively reviewed [86,93,94,95,96,97], and Table 3 summarizes the commonly used methods for EV characterization. Nanoparticle tracking is the most widely used method of EV characterization, which measures EV concentration, size distribution, and particle velocity [104]. Transmission electron microscope demonstrates EV structures that are usually cup-shaped due to dehydration during sample processing [105]. Atomic force microscopy is a technique that provides EV morphology, structural, quantity, and functional analysis at a molecular level. This method also preserves the integrity and bioactivity of EVs [106]. Dynamic light scattering measures particle sizes; however, analysis is limited when various sizes of EV are present, and this cannot be used for functional analysis [104]. Western blot and flow cytometry measure the EV’s surface protein markers and protein cargo compositions [84,85,94]. Flow cytometry can also sort EVs of interest for future functional analysis. The EV’s cargo includes RNA, mRNA, and DNA, which can be detected by next-generation sequencing [84,85,94]. Figure 4 illustrates an EV’s size analyzed by nanoparticle tracking, structure detected by electron microscopy, and surface protein markers measured by Western blot [106]. 

### 4.3. EVs and Neonatal Lung Injury

Although EVs have been characterized in bodily fluids from adults, few studies have identified EVs in the biofluids of neonates [107,108]. Increasing evidence highlights the role of EVs in neonatal lung diseases. EVs have been isolated from a variety of bodily fluids, including TAF [109,110], serum [111], plasma [56], breast milk [112], and amniotic fluid [113] of pre-term infants. Increased AEC-derived EVs were detected in TAF from pre-term infants with severe BPD compared to infants without BPD [109,110]. To characterize EVs during human lung development, Ransom et al. collected tracheal aspirates from premature neonates between 22 and 35 weeks GA and analyzed the EVs by nanoparticle tracking, electron microscopy, and bead-based flow cytometry [110]. EVs were detectable across late canalicular through saccular stages of lung development, with larger sizes of EVs being detected earlier in gestation. EVs contained an abundance of the EV-enriched tetraspanins CD9, CD63, and CD81, as well as epithelial cell and immune cell markers. EVs had increases in select surface proteins (CD24 and CD14) associated with GA and BPD risk. Finally, the expression data obtained from epithelial cells in a single-cell atlas of murine lung development showed that epithelial EV marker expression also changes with developmental time [110]. Together, these data demonstrate an association between EV profile and lung development, and provide a foundation for future functional classification of EVs to determine their role in cell signaling during development and harness their potential as new therapeutic targets in BPD [114]. Infants with severe BPD at 36 weeks PMA compared with age-matched full-term controls had a greater number of EVs in TAFs, but as a group, these EVs were smaller than controls [109]. MiRNA-21 was increased in the serum of pre-term infants born at <32 weeks GA who developed BPD than without BPD [111]. Increased miRNA was also detected in hyperoxia-exposed mouse lung tissues. EVs isolated from umbilical cord venous blood of neonates revealed differential expression of miRNA-17-5p, miRNA-103-3p, miRNA-185-5p, miRNA-20b-5p, miRNA-2001-3p, and miRNA-765 between BPD and non-BPD infants [115]. Among them, miRNA-103-3p and miRNA-185-5p exhibited the most significant reduction, whereas miRNA-2001-3p showed increased expression. Infants with severe BPD also had decreased expression of EV-miRNA-876-3 in their TAF [109]. Interestingly, treating hyperoxia-exposed mice with a miRNA-876-3p mimic resulted in decreased alveolar hyperplasia and neutrophil infiltration [109]. Using a murine model, Genschmer and collaborators studied the function of EVs derived from bronchoalveolar lavage fluid from BPD and non-BPD infants [116]. The results were intriguing, as mice that received intranasal BPD-derived EVs had significant alveolar hypoplasia and right ventricular hypertrophy, suggesting a potential role for EVs in BPD pathogenesis [116]. These findings could have significant implications for our understanding and treatment of BPD.

### 4.4. EVs and Neonatal Brain Injury

In the CNS, each cell type is capable of secreting and taking up EVs, which gives them a vital role in health and during disease. Under physiological conditions, brain cells, including astrocytes, endothelial cells, microglia, oligodendrocytes, and neurons, produce EVs [117,118,119,120,121,122,123]. Some of these EVs mediate brain autocrine and paracrine signaling, including synaptic plasticity, neural trophic support, regulation of myelination, and intercellular communication during brain development [124,125,126]. EVs also play an important role in BBB integrity, and one such example demonstrates that pericyte-derived EVs carry neuroprotective cargo [117]. Additional evidence suggests that EVs derived from the neural progenitor cells improve post-ischemic BBB integrity by enhancing pericyte recruitment via inhibiting the NF-kB pathway and downstream MMP-9 activity, and downregulating expression of ATP binding cassette subfamily member-a (ABCA1) [127]. Under physiological conditions, oligodendrocytes are the myelin-forming cells that can release EVs, which microglia can uptake through micropinocytosis that helps the clearance of oligodendrocyte-derived EV cargo, and this occurs in the absence of microglial activation [120].

Over the last two decades, more knowledge has been gained about the role of EVs in neonatal brain injury in human and experimental models. In 2019, Spaull et al. analyzed the CSF EVs of pre-term infants with post-hemorrhagic hydrocephalus (PHH) and found a heterogeneous size and concentration of EVs between patients [128]. The pathological placental exosomes have been shown to propagate acute and chronic inflammation, leading to brain injury [129]. A study with exosomes isolated from women with pre-eclampsia showed that they disrupt the BBB in vitro and in vivo [130]. In a study conducted by Vechetti and the group demonstrated there was a comparison drawn between the circulation of small EVs in terms of concentration and size between individuals affected by CP as well as typically developed (control group) individuals [114]. CP individuals were seen to present with an overall lower concentration of small EVs. Also, an interesting observation noted in the study was the upregulation of the skeletal muscle-specific miRNA, miR-486, in small EVs from the CP-affected individuals [114]. In animal models of CNS injury, inhibition of CNS EV release reduces systemic responses to CNS inflammation. It also hinders BBB leukocyte infiltration, suggesting a detrimental role of EVs in neuroinflammation [131]. Also, in an IL-1β mouse model of inflammatory brain injury, astrocyte-derived EVs released post-injury can induce a systemic inflammatory response in naïve animals without injury [131]. Microglia-derived EVs are crucial in promoting a proinflammatory microenvironment responding to brain injury [132]. Recent findings suggest that EVs released from microglia in response to brain injury may represent the major signaling pathway of TNF-α secretion, as EV production is markedly induced by activation of the P2X7, P2 receptor X7 by ATP [133]. Activated microglial cells release EVs containing high concentrations of TNF-α, which can cause reactive astrocytic conversion and demyelination [134]. Stimulation of microglia with ATP by activating the P2X7 receptor drastically increases EV release with an increased cargo of IL-1β, IL-6, and TNF-α, inducing a robust inflammatory response [135]. However, it is important to note that EVs also serve as protectors for brain injury. MiRNAs, such as miRNA-182-5p, miRNA-342-3p, and miRNA-92b-3p, are present in hypoxia-preconditioned mouse brain EVs, which protect against apoptosis in hypoxia–ischemia-induced mice [136].

## 5. Inflammasomes and Programmed Inflammatory Cell Death in Pre-Term Lung and Brain Injury 

**Figure 5:**
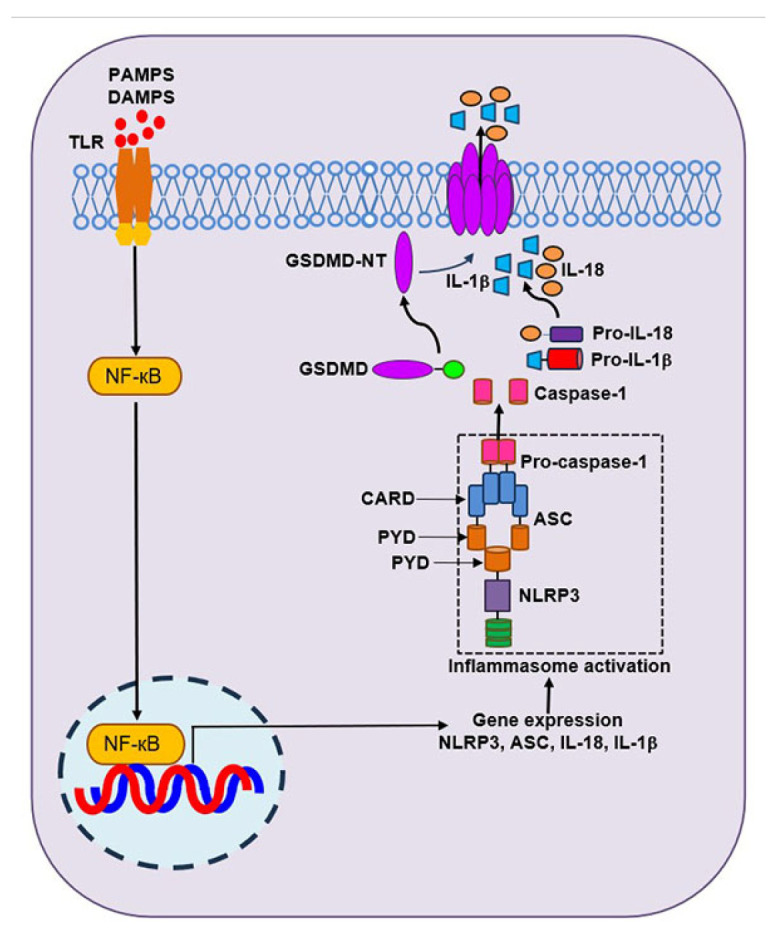
Activation of NLRP3 inflammasome. NLRP3 inflammasome activation requires 2 steps: priming and activation. During the priming step, expression of NLRP3 and other inflammasome components is increased by activation of NF-κB upon PAMPs or DAMPs interaction with toll-like receptors (TLR). In the activation step, NLRP3 is activated by diverse stimuli and the formation of NLRP3 inflammasome that relies on homotypic interaction between the pyrin domain (PYD) and caspase-recruitment domain (CARD). ASC is recruited to cluster PRDs of oligomerized NLRP3 molecules, creating a platform for recruiting the effector caspase-1. The CARD of the procaspase-1 can interact with the aggregated CARDs of ASC, resulting in autolytic cleavage of pro-caspase-1 to P20 and P10 subunits that lead to caspase-1 activation. The activated caspase-1 can cleave and activate GSDMD to release an N-terminal domain that forms membrane pores and leads to pyroptosis. The caspase-1 can also cleave pro-IL-1β and pro-IL-18 to their active forms, IL-1β and IL-18, which can be rapidly released via the GSDMD pores into the extracellular space, leading to inflammation.

Inflammasomes are multiprotein complexes that mediate proteolytic cleavage of GSDMD, pro-IL-1β, and pro-IL-18 by caspase-1 [137,138]. One of the most studied inflammasomes is the NLRP3 inflammasome, which belongs to the nucleotide-binding domain (NBD)- and leucine-rich repeat (LRR)-containing protein (NLP) family [139,140,141,142]. NLRP3 activation requires two steps: a priming step and an activation step (Figure 5). First, NLRP3 expression can be primed by germline-encoded pattern recognition receptors (PRRs), such as TLRs, upon recognition of pathogen-associated molecular patterns (PAMPs) or damage-associated molecular patterns (DAMPs) or by cytokines that engage in immune and inflammatory responses. Upon activation of NF-κB or other transcription factors, the expression of NLRP3 and other inflammasome components is transcriptionally upregulated [139,140,141,142]. Post-translational modifications of NLRP3, including ubiquitination, phosphorylation, and sumoylation, also prime NLRP3 for activation while keeping NLRP3 in an autoinhibited state. In the second step, NLRP3 is activated by diverse microbial and sterile stimuli that often converge to K+ efflux of other ionic changes [139,140,141,142]. The formation of the NLRP3 inflammasome includes three typical components: sensor, adaptor, and effector, and these roles are played by NLRP3, ASC, and caspase-1 [139,140,141,142] (Figure 3). The N-terminal effector domain of NLRP3 consists of a pyrin domain (PYD), 12 leucine-rich repeat (LRR) domains at the C-terminal end, and a central nucleotide-binding oligomerization (NACTH) domain. The ASC comprises an N-terminal PYD domain and a C-terminal caspase-recruitment domain (CARD). Caspase-1 consists of an N-terminal CARD, a large catalytic subunit p20, and a C-terminal small catalytic subunit p10. Assembly of NLRP3 inflammasome relies strictly on homotypic interactions between PYD and CARD. When a ligand is detected, the NLRP3 sensor undergoes a conformational change, releasing itself from its inhibitory state. Through homotypic PYD–PYD interactions, ASC is recruited to cluster PYDs of oligomerized NLRP3 molecules, creating a platform for recruiting effector caspase-1. The CARD of caspase-1 can interact with the aggregated CARDs of ASC, recruiting pro-caspase-1 and rendering caspase-1 fully proteolytical activity. Once locally recruited, pro-caspase-1 increases and undergoes autolytic cleavage to generate p20 and p10 subunits, which form activated caspase-1. The activated caspase-1 can cleave and activate GSDMD to release an N-terminal domain that can bind to the cell membrane and oligomerize to form transmembrane pores. The caspase-1 simultaneously activates pro-IL-1β and pro-IL-18, converting them into mature proinflammatory cytokines. The GSDMD pores allow water and ions to influx, which leads to lytic cell death, a proinflammatory form of programmed cell death known as pyroptosis. The GSDMD pores also allow the release of mature IL-1β and IL-18 into the extracellular space, leading to inflammation [139,140,141,142].

### 5.1. Inflammasomes and Adverse Pregnancy

Growing evidence has shown that physiological and pathological inflammatory processes of pregnancy are related to inflammasome activity. Inflammasome components have been detected during pregnancy in maternal and fetal compartments [143]. The NLRP3 inflammasome is implicated in microbes or danger signals induced by pre-term labor and birth [143,144]. The activation of the NLRP3 inflammasome in the placenta has also been involved in the pathogenesis of pre-eclampsia and other placental disorders [143,145,146]. Antenatal corticosteroids (ACSs) become the standard of care for pregnancies at risk of pre-term labor between 24 and 34 GA. The well-known benefit of ACSs is that they induce rapid fetal lung maturation, manifesting as improved lung compliance and enhanced gas exchange, leading to reduced pulmonary morbidity and improved survival [147]. Some studies found that ACS administration within the optimal time window is associated with elevated cysteine levels, which exerts known antioxidant and anti-inflammatory properties [148]. The anti-inflammatory effect of cysteine is linked to its ability to reduce the expression of NLRP3, ASC, and caspase-1 [148]. In an alarmin-induced pre-term birth mouse model, intra-amniotic injection of alarmin high-mobility group box-1 (HMGB1) reduced gestational length and induced pre-term birth. However, treatment with betamethasone by intra-amniotic injection extended the gestational length and reduced the rate of pre-term birth by 27% [149]. Alarmin plays a crucial role in the activation of inflammasomes, particularly the NLRP3 inflammasome [144]. These data highlight that activation of inflammasomes plays a detrimental role in pre-term labor and birth. 

### 5.2. Inflammasomes in Neonatal Lung Diseases

**Figure 6:**
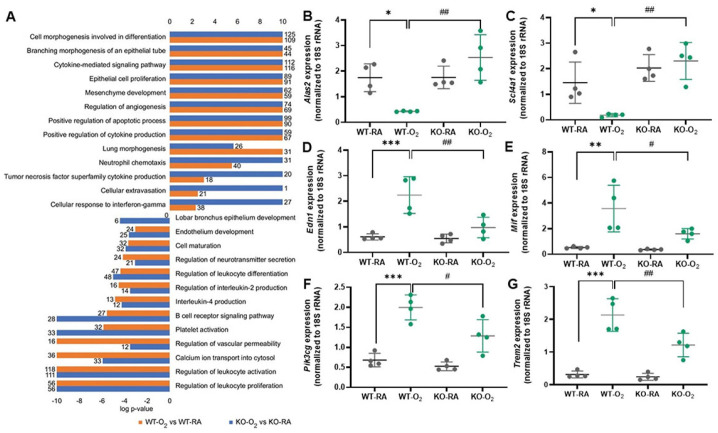
GSDMD-KO reduces hyperoxia modulation of inflammatory, tissue remodeling, and developmental pathways in the neonatal lung. (**A**). Over-representation analysis using Toppcluster to identify similarities and dissimilarities of Gene Ontology terms and pathways modulated by hyperoxia in WT and GSDMD-KO lungs. Bars represent the log p-value, and the number of genes associated with each term is displayed at the end of the bar. In GSDMD-KO lungs, genes induced by hyperoxia were more strongly associated with TNF superfamily cytokine production, cellular extravasation, and cellular response to IFN-g, while suppressed genes in GSDMD-KO were uniquely associated with lobar bronchus epithelium development and B cell receptor signaling pathways. n = 3 animals/group. qRT-PCR validation of differentially expressed genes between hyperoxia-exposed WT and hyperoxia-exposed GSDMD-KO lungs included *Alas2* (**B**), *Scl4a1* (**C**), *Edn1* (**D**), *Mif* (**E**), *Pik3cg* (**F**), and *Trem2* (**G**). n = 4/group. * *p* < 0.05, ** *p* < 0.01, *** *p* < 0.001, WT-O_2_ vs. WT-RA. ^#^ *p* < 0.05, ^##^ *p* < 0.01, WT-O_2_ vs. KO-O_2_. Ref. [55] with permission.

Liao et al. reported in 2015 that activation of the NLRP3 inflammasome is associated with the development of BPD [150]. They showed that exposure to 85% hyperoxia in neonatal mice from P3 to P14 increased caspase-1 activation, IL-1β, inflammation, and decreased alveolarization. They further demonstrated that NLRP3 KO mice, when exposed to 85% oxygen, show no caspase-1 activity, no IL-1β, and no inflammatory response and undergo normal alveolarization. Additionally, treating hyperoxia-exposed mice with either an IL-1 receptor (IL-1r) antagonist to block IL-1β or glyburide to block the NLRP3 inflammasome showed promising results in reducing inflammation and increasing alveolarization. This is particularly significant given the predictive potential of the IL-1β/IL-1ra ratio in tracheal aspirates from pre-term infants with respiratory failure for the development of BPD. This important study demonstrates the critical role of NLRP3 in BPD pathogenesis in experimental models and clinical investigations. While caffeine is commonly used in pre-term infants for apnea, in this hyperoxia-induced BPD model, treatment with caffeine significantly reduced oxidative stress, promoted alveolar development, and attenuated inflammatory infiltration and lung injury, and these were associated with a significant inhibition of NLRP3 inflammasome protein and NF-κB pathway [151]. In another newborn mouse model of hyperoxia-induced lung injury, treatment with acetate significantly reduced the expression of TNF-α, IL-1β, IL-18, NLRP3, and caspase-1 [152]. Tert-butylhydroquinone (TBHQ), an inhibitor of Nuclear factor e2-related factor 2 (Nrf2), was shown to reduce NLRP3 inflammasome activation, decrease IL-1β and IL-18 expression and activation, as well as inhibit pyroptosis in the lungs of hyperoxia-induced newborn mice [153]. In neonatal rats exposed to hyperoxia, inhibition of Rac1, a member of the Rho GTPase family with NSC23766, significantly decreased NLRP1 inflammasome activity, reduced lung macrophage infiltration, and improved alveolar and vascular development [154]. Vaidya et al. showed that recombinant cysteine-rich protein 61 (CCN1) reduced macrophage and neutrophil infiltration, decreased NLRP1 inflammasome activation, and improved alveolar and vascular development in hyperoxia-exposed newborn rat lungs [155]. Also, in a hyperoxia-induced BPD rat model, 18b-Glycyrrhetinic acid treatment inhibited the activation of NF-κB and the NLRP3 inflammasome, decreased ROS level and pulmonary inflammation, improved alveolar development, and increased body weight of neonatal rats exposed to hyperoxia [156]. Simvastatin inhibited NLRP3 inflammasome activation and ameliorated lung injury in hyperoxia-induced BPD via Kruppel-like factor 2 (KLF2)-mediated mechanism [157]. The direct evidence that GSDMD plays an important role in hyperoxia-induced BPD was provided by studies demonstrating that GSDMD-KO protects newborn mice from hyperoxia-induced BPD by reducing macrophage infiltration, improving alveolarization and vascularization, and decreasing cell death [55]. Furthermore, RNA-seq analysis has revealed that GSDMD-KO significantly modulated hyperoxia-induced transcriptomic responses in the lung (Figure 6) [55]. In GSDMD-KO lungs, hyperoxia-induced genes were strongly associated with TNF superfamily cytokine production, cellular extravasation, and cellular response to IFN-g. In contrast, hyperoxia-suppressed genes were uniquely associated with lobar bronchus epithelium development and B cell receptor signaling pathways compared to hyperoxia-exposed wildtype (WT) lungs. Some of the differentially expressed genes between hyperoxia-exposed WT and hyperoxia-exposed GSDMD-KO lungs included mitochondrial erythroid-specific 5-aminolevulinate synthase 2 (*Alas2*), solute carrier family 4 member 1 (*Slc4a1*), endothelin 1 (*Edn1*), macrophage migration inhibitory factor (*Mif*), phosphatidylinositol 3-kinase catalytic subunit gamma (*Pik3cg*), and triggering receptor expressed on myeloid cells 2 (*Trem2*). These genes are important in regulating pulmonary vascular remodeling (*Alas2* and *Scl4a1*), inducing lung fibroblast proliferation (*Edn1*), controlling both innate and adaptive immune responses (*Mif*), initiating inflammatory responses (*Pik3cg*), and driving gene expression programs involved in phagocytosis (*Trem2*). The potential implications of these findings are intriguing and warrant further investigation. 

### 5.3. Inflammasomes in Neonatal Brain Injury

The role of inflammasomes in neonatal brain injury was abundantly studied in experimental models of hypoxic–ischemic encephalopathy (HIE). In 2018, Chen et al. showed that hypoxic–ischemic brain injury significantly increased the expression of inositol requiring enzyme-1 alpha (IRE1α) in the brain. Intranasal administration of STF-083010, an IRF1α inhibitor, reduced brain injury, improved neurological behavior, and improved expression of MiR-17-5p. Meanwhile, administration of miR-17-5p mimic reduced NLRP3 inflammasome activation, caspase-1 cleavage, and IL-1β production, as well as brain infarct volume [158]. Serdar et al. showed that LPS pre-sensitization significantly increases brain area loss and induces microglia activation and neuronal injury after mild hypoxia–ischemia. They also found that microglia upregulate proinflammatory genes involving NLRP3 inflammasome [159]. Lv et al. found that in HIE patients, the elevation levels of the pyroptotic pathway tightly correlate with the severity of HIE. Treatment with MCC950, a small molecule inhibiting NLRP3 inflammasome and thus pyroptosis, alleviated pyroptosis and injury severity in rats with neonatal hypoxic–ischemic brain damage [160]. Increasing evidence indicates that miRNAs are involved in the process of HIE, and miR-374a-5p is downregulated in HIE patients. Further, overexpression of miR-374a-5p significantly attenuated brain injury and inhibited the release of proinflammatory cytokines in neonatal rat HIE models. In vitro, miR-347a-5p inhibited LPS-induced microglial proinflammatory cytokine production by regulating NLRP3 inflammasome [161]. Few studies focused on the roles of inflammasomes in brain white matter injury (WMI). Caffeine has been shown to inhibit NLRP3 inflammasome activation, reduce expression of IbaI, an active microglial marker, inhibit microglia M1 polarization, promote microglia M2 polarization, and improve long-term cognitive function in neonatal rats with hypoxic–ischemic WM disease [162]. Diallyl disulfide (DADS) is an allicin extract with detoxifying, antibacterial, and cardiovascular disease-protective effects. It was tested in HIE-induced brain damage in rats and showed DADS significantly reduced the cerebral infarct volume, alleviated inflammatory reaction, reduced astrocyte activation, promoted tissue structure recovery, and improved pyroptosis caused by HIE [163]. Our novel research on the effects of GSDMD-KO in newborn mice under oxygen exposure has brought forth intriguing findings. When GSDMD-KO and WT newborn mice were exposed to 85% oxygen, GSDMD-KO mice demonstrated significant resistance to oxygen. They showed decreased pyroptosis and increased proliferation compared to WT brains. Furthermore, GSDMD- KO also prevented gene expression associated with key pathways regulating neuronal and vascular development and differentiation, axonogenesis, glial cell differentiation, hypoxia-induced factor 1 signaling, and neuronal growth factor pathways [164]. These findings, unique in their implications, pave the way for further exploration in the field.

### 5.4. EV–Inflammasomes Mediate Lung–Brain Axis in Neonatal Brain Injury

**Figure 7:**
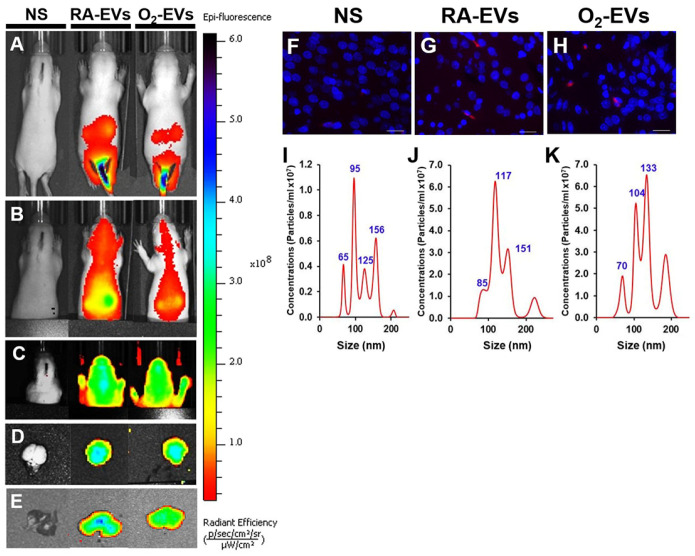
Adoptively transferred circulating EVs track to the lung and brain of normal neonatal rats. EVs isolated from the plasma of room air-maintained (RA) or hyperoxia-exposed (O_2_) rats were labeled with Exo-Glow and adoptively transferred into normal neonatal rats by intravenous injection. As illustrated in (**A**–**C**), both Exo-Glow labeled RA-EVs and O_2_-EVs rapidly distributed throughout the body and were localized in both lung and brain at 1 and 4 h after tail vein injection. Their homing to the brain (**D**) and lung (**E**) tissues was further confirmed by ex vivo imaging after dissection at 4 h. To determine if circulating EVs can cross BBB and are present in brain tissue for longer than 4 h, Dil-dye labeled EVs were similarly injected via tail veins, and at 24 h, EVs were isolated from the CSF and examined in brain tissues. The Dil signals were detected in the brain tissue sections of rats injected with both RA-EVs and O_2_-EVs but not from sham animals (**F**–**H**). Magnification: 20×. Scale bars: 50 μm. In addition, high concentrations of CSF EV particles were detected in animals that received either RA-EVs ((**J**), 17.55 ± 1.9 × 10^7^, n = 2 pooled of 3 CSF, *p* < 0.01) or O_2_-EVs ((**K**), 26.7 ± 16.6 × 10^7^, n = 2 pooled of 3 CSF, *p* < 0.05), compared to the sham animals ((**I**), 4.95 ± 0.57 × 10^7^, n = 4 pooled of 3 CSF). Overall, these results confirm that hyperoxia-induced circulating EVs can cross BBB and be taken up by brain cells. Ref. [56] with permission.

Several studies were performed in experimental models to answer whether EV–inflammasomes mediate the lung–brain axis in neonatal brain injury. The critical roles of circulating EVs released from hyperoxia-exposed and mechanically ventilated newborn rats in inducing brain injury were investigated in healthy newborn rats [56,165]. The hyperoxic model was established by exposing newborn rats to room air or 85% oxygen for two weeks, and circulating EVs were isolated from the rats’ plasma [56]. The EVs were analyzed using nanoparticle tracking, fluorescence-activated cell sorting (FACS), and Western blot. It was found that the EVs from hyperoxia-exposed rats contain increased levels of both surfactant protein C (SPC), an AT2 cell marker, and GSDMD, a key executor of inflammasome-induced cell pyroptosis, suggesting that these GSDMD+ EVs may be produced by AT2 cells. In order to study the roles of these EVs in brain injury, adoptive transfer experiments were performed by injecting Exo-Glow-labeled EVs via the tail veins into healthy newborn rats, and surprisingly, these labeled EVs were detected in the lungs and brain of adoptively transferred rats (Figure 7). A similar experiment with Dil-labeled EVs showed that Dil labeling was detected in the brain tissues, and CSFs from EV-injected animals had higher concentrations of EVs than sham animals (Figure 7). Subsequent adoptive transfer experiments showed that rats receiving EVs from the hyperoxia-exposed rats induced lung inflammation, signified by increased inflammatory cell infiltration in the alveolar airspaces, accompanied by increased expression of inflammatory cytokines and chemokines. Furthermore, the rats that received the EVs from hyperoxia-exposed rats had drastically reduced alveolarization and vascular density. In vitro experiments by treating the pulmonary vascular endothelial cells (PVECs) with EVs from RA and hyperoxia-exposed rats demonstrated that the PVECs treated with the EVs from hyperoxia-exposed rats had increased cell death and reduced cell survival [56]. Moreover, the EVs from hyperoxia-exposed rats had detrimental effects on the receipt rats’ brains. The brains of these rats showed microglial activation and increased expression of proinflammatory cytokines, indicators of inflammation. These brains also had increased cell death in the cortex, subventricular, and subgranular zones. Additionally, in vitro experiments in neural stem cells (NSCs) demonstrated that EVs from hyperoxia-exposed rats increased cell death and decreased cell survival [56]. Furthermore, EVs from cultured hyperoxia-exposed lung epithelial cells induced pyroptosis in NSCs [56]. These data revealed a potential role of lung epithelial-derived circulating EVs in mediating novel lung–brain crosstalk that is critical in both neonatal lung and brain injury. 

Given injurious mechanical ventilation is associated with neonatal lung and brain injury, Chavez et al. investigated the role of EVs in mediating lung–brain crosstalk in mechanical ventilation-associated brain injury in newborn rat models [165]. This study demonstrated that injurious mechanical ventilation with higher tidal volumes induced inflammasome activation, represented by increased activated caspase-1, IL-1β, and GSDMD in both lung and brain and induced microglial activation and cell death in the brain [165]. It was found that the circulating EVs isolated from the neonatal rats with ventilator-induced lung injury had increased caspase-1. The adoptive transfer of these EVs into healthy newborn rats resulted in neuroinflammation with microglial activation and increased activation of caspase-1 and GSDMD in the brain. These pathological changes were similar to those observed in neonatal rats that received injurious mechanical ventilation [165]. Thus, circulating EVs may contribute to brain injury and possibly poor neurodevelopmental outcomes in pre-term infants exposed to hyperoxia and mechanical ventilation [165].

**Figure 8:**
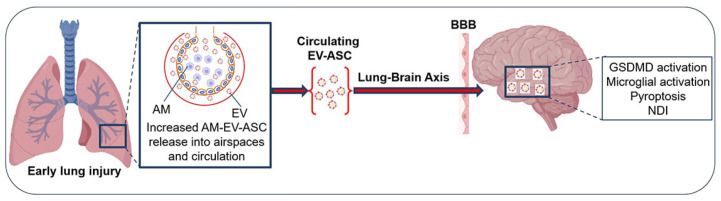
EV-inflammasome mediated lung-brain axis. The alveolar macrophages (AMs) in early injured lungs release EVs that contain an increased cargo of ASC. These EVs contribute to BPD pathogenesis by inducing lung inflammation and inhibiting alveolarization and vascularization. These EVs are released to the circulation, cross the BBB, and are taken up by neural cells. The ASC cargo can activate GSDMD in specific neural cells and result in brain injury by activating microglial cells and inducing cell death, possibly through pyroptosis mechanisms. The molecular and cellular changes can lead to long-term neurodevelopmental impairment (NDI). Ref. [106] with permission.

Most recently, we published a study that examined the EVs isolated from the plasma of pre-term infants at risk for BPD at 7 days of life [106]. We found that the EVs from infants who were on higher oxygen therapy (≥30%, HO_2_) had increased levels of alveolar macrophage-derived EV-ASC compared to infants on lower oxygen therapy (<30%, LO_2_). To assess the function of these EVs, we performed adoptive transfer experiments by injecting them into the circulation of newborn mice and examining the lungs and brains on P17. We discovered that mice that received EVs from the HO_2_ patients had increased lung inflammation, decreased alveolarization, and disrupted lung vascular development, the hallmarks of BPD. Importantly, these EVs crossed the BBB, and the EVs from infants on HO_2_ caused inflammation, reduced cell survival, and increased cell death with the feature of pyroptosis in the hippocampus [106]. These data support a novel AM-derived EV–inflammasome model that mediates the lung–brain axis, which leads to brain inflammatory injury (Figure 8). These studies provide experimental and clinical evidence for EV–inflammasomes acting as novel mediators for lung injury-associated brain injury.

## 6. Clinical Implications of EV-Inflammasomes in BPD and Bain Injury

In comparison to the extensive investigations of EV-inflammasomes in experimental BPD and brain injury, the clinical implications of EV-inflammasomes in BPD and brain injury in preterm infants are less studied. However, EV-inflammasomes have great potential to serve as biomarkers and be used as novel therapeutics for preterm infants with BPD and brain injury. 

Few studies have evaluated the cargos of EVs isolated from the tracheal aspirates and serum of preterm infants at risk for developing BPD. EVs isolated from TAF of preterm infants with severe BPD had higher numbers of EVs but were smaller in seizes than EVs from term control infants [109]. The most reduced exosome cargo was miR-876-3p. A study of EVs in TAF from preterm infants born between 22 and 35 weeks of gestational age demonstrated that EVs from infants with a risk of BPD had increased surface proteins of CD24 and CD14 [110]. These proteins are typically expressed by immune cells and epithelial cells. EVs isolated from the serum of preterm infants have increased miR-21 in BPD patients at 28 days of life compared to non-BPD patients [111]. Preterm infants developing BPD also had higher levels of macrophage-derived EVs in their plasma at 7 days of life [106]. There is evidence that inflammasomes are biomarkers for BPD and brain injury in preterm infants. Liao et al. demonstrated that increased IL-1b:IL-1R in TAF of preterm infants in the first 3 days of life predicts BPD at 36 weeks postmenstrual age [150]. The ELGAN study found that preterm infants on more extended mechanical ventilation (14 days) had elevated blood concentrations of IL-1β and IL-18 and other cytokines and chemokines compared to those on shorter mechanical ventilation (<7 days) [166]. A recent study discovered that Hsa_circ_0001359, a circRNA, differentially expressed between the BPD infants and the non-BPD infants at 1 week, 2 weeks, and 4 weeks of age. Further, the expression of hsa_circ_0001359 at 1 week of age had a higher predictive value for BPD development [167]. However, none of these studies linked these potential biomarkers to neurodevelopmental outcomes. It is important to determine if lung cell-derived EV-inflammasomes can serve as biomarkers not only for BPD but also for brain injury and long-term neurodevelopmental outcomes.

One important question is whether we can target EVs and inflammasomes to prevent and treat BPD and brain injury in pre-term infants. Postnatal steroids as anti-inflammatory agents are commonly used in BPD patients who are on prolonged invasive mechanical ventilatory support, and they are tested in animal models with neonatal lung injury [166,167]. It is known that glucocorticoids elicit their anti-inflammatory effects by inhibiting IL-1 and IL-1 receptor antagonist (IL-1ra) expression [168]. Glucocorticoids also inhibit cytokine-induced pro-inflammatory transcription factors, such as NF-κB, activator protein (AP)-1, and signal transducers and activators of transcription (STATs) [168]. Given that NF-κB and IL-1 are involved in the inflammasome cascade (Figure 3), reducing NF-κB and IL-1 by corticosteroids suggests their role in inhibiting the inflammasome pathway. A recent systemic review of 39 randomized clinical trials (RCTs) found that postnatal corticosteroid therapies, including dexamethasone and hydrocortisone, are beneficial for reducing BPD risk, decreasing the duration of invasive mechanical ventilation, and reducing supplemental oxygen therapy [169]. Early and higher doses of dexamethasone are more effective for respiratory outcomes, but they cause systemic hypertension and hyperglycemia. Hydrocortisone has lesser effects on respiratory outcomes, systemic hypertension, or hyperglycemia. One of the earlier studies showed dexamethasone therapy increased the incidence of CP [170]. Another study found at school age, children who were treated with dexamethasone during the neonatal period had significantly reduced motor coordination, motor skills, visual–motor integration, and remarkably lower full IQ, verbal IQ, and performance IQ scores [171]. Most of these clinical studies did not measure inflammasome pathways, and the effects of postnatal steroids on lung and brain structure remain unknown. However, a recent systemic review of the impact of postnatal corticosteroids on lung development in animal models found that postnatal corticosteroids persistently decreased body weight and caused alveolar simplification [167]. Overall, postnatal steroids are one of the few anti-inflammatory drugs that are continuously used in pre-term infants at risk for developing BPD. 

## 7. Perspective

Many pharmacological agents are being investigated on their properties to inhibit EV release as research tools and therapeutic modalities in adult patients and animal models. Some of these agents inhibit EV trafficking, such as calpeptin [172], manumycin A [173], and Y27632 [174]; others inhibit lipid metabolisms, such as D-pantethine [175], imipramine [133], and GW4869 [176]. Additional drugs that inhibit various kinases have also been tested, including bisyndoylmaleimide I (protein kinase C inhibitor) [177], U0126 (inhibitor of MEK 1 and MEK 2, two protein kinases belonging to the mitogen-activated protein kinase family) [178], and clopidogrel (anticoagulant) [179]. Overall, these agents are mainly tested in experimental models of cancer cells [180]. We have a long way to go to identify effective anti-EV agents for preventing and treating BPD and brain injury in pre-term infants. In contrast to anti-EV therapy, anti-inflammasome therapy is more promising for preventing and treating BPD and brain injury in pre-term infants. Many of the studies that involve anti-inflammasome drugs in experimental models of BPD and brain injury have been reviewed in the above sections. Chiarini et al. summarized an extensive list of specific and non-specific NLRP3 inhibitors tested in brain disorders [181]. VX765 is a specific caspase-1 inhibitor that has been approved by the FDA to be used in clinical trials of adult patients with psoriasis (ClinicalTrials.gov Identifier: NCT00205465) and epilepsy (ClinicalTrials.gov Identifier: NCT01048255 and NCT01501383). Disulfiram inhibits GSDMD pore formation associated with pyroptosis, which the FDA also approves for treating chronic alcoholism [182,183]. GDC-2394 is an NLRP3 inhibitor approved by the FDA for the first-in-human phase I trial in normal volunteers [184]. Anakinra, a nonglycosylated recombinant version of human IL-1Ra, is used clinically for rheumatoid arthritis and cryopyrin-associated periodic syndrome [185]. Tadekinig alfa, a recombinant version of human IL-18 binding protein, is currently being investigated in a single-arm, open-label phase 3 trial in patients with NLRC4-associated hyperinflammation [186]. Clearly, targeted therapies for EVs and inflammasomes are underdeveloped for BPD and brain injury in pre-term infants. It is imperative that some of these therapies be tested in pre-term infants with BPD and brain injury in future clinical investigations. 

## 8. Conclusions

EVs and inflammasomes are emerging targets in clinical and experimental neonatal lung and brain injury. We presented the evidence for BPD as an important risk factor for neonatal brain injury and long-term NDI. We discussed how EVs and inflammasomes work under physiological conditions and, more importantly, in lung and brain injury of pre-term infants. We presented abundant data demonstrating the critical role of EVs and inflammasomes in regulating lung and brain injury in experimental models, particularly in hyperoxia-induced BPD and brain injury, mimicking clinical scenarios in the neonatal intensive care unit. Intriguingly, lung-derived EVs in rodents and pre-term infants carry inflammasome cargo, which can cross the BBB, be taken up by brain cells, and induce brain inflammatory injury. In contrast to many studies investigating the therapeutic role of EVs from various stem cells in neonatal lung and brain injury, this review focused on the novel role of EVs in inducing neonatal lung and brain injury. Targeting the EV–inflammasome cascade may have a great potential for treating and preventing pre-term infants at risk for developing BPD and brain injury.

## Figures and Tables

**Table 1:** Definitions of BPD.

2001 NICHD workshop definition (reference [2])
Need oxygen supplement for at least 28 days at 36 weeks’ PMA: ▪ Mild BPD: FiO_2_ = 0.21 ▪ Moderate BPD: FiO_2_ < 0.3 ▪ Severe BPD: FiO_2_ ≥ 0.3 and/or CPAP and/or IMV
2017 Canadian definition (Reference [22])
Evaluating infants for oxygen and/or respiratory support at 40 weeks’ PMA may increase the predictive ability of respiratory outcomes.
2018 revised NICHD definition (Reference [26])
Determine BPD severity by the mode of respiratory support and FiO_2_ required at 36 weeks’ PMA: ▪ Mild BPD (grade I)—any of the following: Hood O_2_ with FiO_2_ 0.22 to 0.29 Nasal cannula at <1 L/min with FiO_2_ 0.22 to 0.70 Nasal cannula at 1 to <3 L/min with FiO_2_ 0.22 to 0.29 CPAP, NIPPV, or nasal cannula (including HFNC) ≥ 3 L/min with FiO_2_ 0.21 ▪ Moderate BPD (grade II)—any of the following: Hood O_2_ with FiO_2_ ≥ 0.30 Nasal cannula at <1 L/min with FiO_2_ ≥ 0.70 Nasal cannula at 1 to <3 L/min with FiO_2_ ≥ 0.30 CPAP, NIPPV, or nasal cannula (including HFNC) ≥ 3 L/min with FiO_2_ 0.22 to 0.29 Invasive mechanical ventilation with FiO_2_ 0.21 ▪ Severe BPD (grade III)—either of the following: CPAP, NIPPV, or nasal cannula ≥ 3 L/min with FiO_2_ ≥ 0.30 Invasive mechanical ventilation with FiO_2_ > 0.21
2019 NICHD (Jenson) definition (reference [27])
Define BPD severity according to the mode of respiratory support administered at 36 weeks’ PMA, regardless of supplemental oxygen use: ▪ Mild BPD (grade I): Requires low-flow nasal cannula (≤2 L/min) ▪ Moderate BPD (grade II): Requires CPAP, NIPPV, or nasal cannula flow of >2 L/min (including HFNC) ▪ Severe BPD (grade III): Requires invasive mechanical ventilation

NICHD: National Institute of Child Health and Human Development; PMA: postmenstrual age CPAP: continuous airway pressure; NIPPV: noninvasive intermittent positive pressure ventilation; HFNC: high-flow nasal cannula; FiO_2_: fraction of inspired oxygen; O_2_: oxygen. UpToDate 15 August 2024, with permission.

**Table 2:** Exosome isolation methodology.

Methods	Pros	Cons	References
Ultracentrifugation	Gold standard Cost-effective Allowing large, simple quantification	Disruption of exosome structure by high shear forces Exome loss, fusion, deformation Co-isolation of contaminatinants	[86,94,99]
Ultrafiltration	Simple method More rapid than ultracentrifugation	Exosome damage due to shear stress Exosome loss due to membrane adhesion and blockage	[86,94,100]
Size-exclusion chromatography	High yield Economical and nondestructive Maintaining vesicle integrity and biological function	Labor intensive Low purity Specialised equipment and columns used are expensive	[86,95,101,102]
Immunoaffinity-based capture	High purity and specificity Gentle procedure—maintains the function of exosomes after purification	Low yield Only isolate subtypes of exosomes with positive markers Expensive cost of high-affinity antibodies Can only be used with lower sample volumes Long time procedure	[86,95]
Microfluidics-based techniques	Fast separation High purity Allow exosome isolation and characterization simultaneously	Low sample capacity Need complicated equipment Expensive in device development	[86,103]

**Table 3:** Exosome characterization methods.

Methods	Procedure and Advantages	Disadvantages	References
Nanoparticle tracking analysis	One of the most commonly used methods Detecting particle sizes 50–1000 nm Provide particle concentrations	Difficult to distinguish contaminated protein from exosomes	[104]
Electron microscope (scanning electron microscope and transmission electron microscope)	Directly observe the morphology of exosomes Require a small amount of samples	Loss of exosomes during extensive sample preparation Shape modifications through sample preparation	[105,106]
Atomic force microscopy	Detects exosome morphology in 3D space Generates 3D topography images of the exosomes with a resolution limit of around 1 nm Requires a small sample amount	Measuring exosomes in their natural states The conditions of temperature, humidity, pressure, varying scan speed, and force between tip and sample cause variation in measurements	[86,94]
Dynamic light scattering	Determination of different size distributions of particles Detecting particle sizes ranging 1–10,000 nm Small sample volumes required	Challenging in the detection of smaller particles	[86,94]
Western blot	Qualitatively and quantitatively analyzing protein markers High detection sensitivity and specificity	Time consuming Less amenable for high throughput adaptation	[84,85,94]
Flow cytometry	Detecting particle sizes ranges 200–500 nm Utilizing particle surface protein expression patterns High detection sensitivity and specificity	Long procedure Unable to detect particles at <200 nm	[84,85,94]
RNA content	High-throughput RNA-seq Validating specific genes by qRT-PCR	Low yield of RNAs Limitation in subsequence quantification methods	[84,85,94]

## Data Availability

Not applicable.
